# Effects of propofol and inhaled anesthetics on postoperative complications for the patients undergoing one lung ventilation: A meta-analysis

**DOI:** 10.1371/journal.pone.0266988

**Published:** 2022-10-20

**Authors:** Jing Yang, Qinghua Huang, Rong Cao, Yu Cui

**Affiliations:** Department of Anesthesiology, The Affiliated Hospital, School of Medicine, UESTC Chengdu Women’s & Children’s Central Hospital, Chengdu, China; Universidad de Chile, CHILE

## Abstract

**Introduction:**

With the widespread use of one-lung ventilation (OLV) in thoracic surgery, it is unclear whether maintenance anesthetics such as propofol and inhaled anesthetics are associated with postoperative complications. The purpose of this study was to compare the effects of propofol and inhaled anesthetics on postoperative complications in OLV patients.

**Methods:**

PubMed, EMBASE, Medline, and Cochrane Library were searched for relevant randomized controlled trials until 09/2021. All randomized controlled trials comparing the effect of propofol versus inhaled anesthetics on postoperative complications in OLV patients were included. All randomized controlled trials comparing:(a) major complications (b) postoperative pulmonary complications (c) postoperative cognitive function (MMSE score) (d) length of hospital stay (e) 30-day mortality, were included.

**Results:**

Thirteen randomized controlled trials involving 2522 patients were included in the analysis. Overall, there was no significant difference in major postoperative complications between the inhaled anesthetic and propofol groups (OR 0.78, 95%CI 0.54 to 1.13, p = 0.19; I^2^ = 0%). However, more PPCs were detected in the propofol group compared to the inhalation anesthesia group (OR 0.62, 95%CI 0.44 to 0.87, p = 0.005; I^2^ = 37%). Both postoperative MMSE score (SMD -1.94, 95%CI -4.87 to 0.99, p = 0.19; I^2^ = 100%) and hospital stay (SMD 0.05, 95%CI -0.29 to 0.39, p = 0.76; I^2^ = 73%) were similar between the two groups. The 30-day mortality rate was also not significantly different between groups (OR 0.79, 95%CI 0.03 to 18, p = 0.88; I^2^ = 63%).

**Conclusions:**

In patients undergoing OLV, general anesthesia with inhaled anesthetics reduced PPC compared to propofol, but did not provide clear benefits on other major complications, cognitive function, length of hospital stay, or mortality.

## Introduction

According to the literature, approximately 3% of surgical patients develop severe complications and 0.4% die postoperatively [1]. And lung cancer is the leading reason of cancer-related death in the United States [2]. One-lung ventilation (OLV) has become a necessary technique in thoracic surgery because it facilitates surgery and prevents contamination of the other lung [3]. However, one-lung ventilation increases the risk of postoperative complications by potentially causing ischemia and hypoxia in the nonventilated lung, pressure trauma and excess fluid in ventilated lung tissue, and alveolar and systemic inflammatory responses [4]. The incidence of postoperative pulmonary complications (PPC) is much higher in patients operated on with OLV than in those without [5].

Christopher, et al. found that in cardiac surgery, inhalation anesthesia was associated with a significant outcome advantage and lower mortality [6]. However, Bassi, A [7] and Modolo, NS [3] found little evidence from randomized controlled trials (RCTs) in 2008 and 2013 that showed significant differences in specific postoperative outcomes between general anesthesia maintained with inhalation and intravenous anesthesia such as propofol in the case of OLV. Subsequently, several RCTs and systematic reviews have suggested that inhaled anesthesia may preserve cardiac function, decrease PPC, and attenuate local alveolar inflammatory responses in patients undergoing OLV [8–10].

Since 2013, more and more clinical RCTs have been published examining the effects of different sedative anesthetics on major complications in OLV patients. Therefore, we conducted this meta-analysis to compare the effects of inhaled anesthetics (Sevoflurane or Desflurane) and propofol on postoperative outcomes.

## Methods

We followed the recommendations of the Cochrane Handbook for the Systematic Review [11]. The meta-analysis is also registered at https://www.crd.york.ac.uk/prospero / under No. CRD420202222856.

### Retrieval strategy

Two authors (JY, QHH) separately searched Pubmed, Medline, Embase, and Cochrane Central registers for relevant RCTs from January 1, 2000, to September 31, 2021. Searches were performed using various combinations of keywords and MeSH terms. The search terms are listed in Table 1, and the search was limited to the English language.

**Table 1:** The specific keywords and MeSH terms during the screening process.

anesthesia, intravenous	anesthetics, inhalation	one lung ventilation	RCT
intravenous anesthesia	anesthetic gases	single lung ventilation	Randomized controlled trial
intravenous anesthetics	inhalation anesthetics	Ventilation, One-Lung	Controlled clinical trial
intravenous anesthetic agent	inhalation anesthesia	Ventilation, Single-Lung	Randomized
propofol	inhaled anesthesia	lung separation techniques	Randomly
Diprivan	volatile anesthetics	Separation Technique, Lung	Trial
Disoprofol	sevoflurane	Technique, Lung Separation	
	sevorane	Lobectomy	
	desflurane	thoracic surgery	
	isoflurane		

We used Boolean operator “OR” to search every potentially eligible article that was relevant to Intravenous anesthetics/ Inhaled anesthetics/ One lung ventilation. Then, we used the operator “AND” to combine the above results to accomplish the screening process.

### Inclusion criteria

1. Population: Patients (>18 years old) scheduled for standby thoracic surgery under OLV.
2. Intervention: Patients who maintained anesthesia with inhaled anesthetics during OLV.
3. Comparison: Patients received propofol to maintain anesthesia during OLV.
4. Outcomes:

The primary endpoint was the occurrence of major complications assessed by Clavien-Dindo score (grade III to V) or assessed by surgeon (complications that needs more intensive treatments including overall cardiac events, myocardial infarction, acute renal failure, hepatic failure, disseminated intravasal coagulation, extrapulmonary infection, gastrointestinal failure, coma).

The secondary evaluation items were the number of PPC (hypoxemia, acute respiratory distress syndrome, pulmonary infiltrates, pneumonia, pleural effusions, atelectasis, pneumothorax, bronchospasm, cardiopulmonary edema, aspiration pneumonitis); the scores of Mini-mental State Examination (MMSE) during hospital admission, length of hospital stay and 30-day mortality.

(5) Study: Randomized controlled studies.

All trials in which the population, intervention, comparison, study, and at least one outcome were reported as described above were included.

#### Exclusion criteria

Duplicate studies, non-human or pediatric studies, conference abstracts, studies published before the 2000s, and studies from which data could not be extracted.

#### Data extraction

Based on the above criteria, two authors (JY, QHH) sequentially enrolled in the study and independently extracted data: publication information (first author name, year of publication), participant characteristics (sample size, type of surgery, anesthesia induction scheme, OLV and operation time, OLV strategy) and outcome information. Disagreements regarding eligibility between the two investigators were resolved by discussion. If necessary, a third researcher (RC) was involved in making a determination. Data were extracted or calculated from figures and tables using the Engauge Digitizer 5.1 program (M. Mitchell, Engauge Digitizer, http://digitizer.sourceforge.net) as needed. All extracted data were collected in standardized Excel files by the two authors and double-checked by YC for accuracy.

### Bias risk assessment and strength of evidence

Two reviewers (JY, QHH) independently assessed the methodological quality of the included trials using methods recommended by the Cochrane Collaboration. For each trial, the criteria used to assess quality were random sequence generation, allocation concealment, performance bias, detection bias, attribution bias, reporting bias, and others. Each criterion was categorized as "yes," "no," or "unclear," and a simple rating for each trial was classified into three levels (low risk of bias, unclear risk of bias, and high risk of bias). The Grading of Recommendations, Assessment, Development and Evaluations approach (GRADEpro; gdt.gradepro.org) was approved to comprehensively assess the quality of evidence for each outcome. In this approach, each outcome begins as high-quality evidence, but may be downgraded by one or more of five categories of limitations (risk of bias, inconsistency, indirectness, imprecision, and reporting bias). Finally, this approach depicted the apparent quality of each outcome as low, moderate, or high.

### Statistical analysis

According to DerSimonian and Laird method performed by Review Manager 5.3 (RevMan, The Cochrane Collaboration, Oxford, UK), differences were expressed as risk ratio (RR) with 95% confidence intervals (CI) for dichotomous data, and the differences between continuous data were expressed as mean differences (MDs) or standardized mean differences (SMD) with 95% CI. Due to the small number of trials and high heterogeneity among trials, data pooled by five or fewer trials or with heterogeneity values greater than 50% were further subjected to random effects measurement using the Hartung-Knapp-Sidik-Jonkman (HKSJ) method. Since Joanna, et al. [12] found that the HKSJ method was proved superior to the DerSimonian-Laird method in meta-analyses with a smaller number of trials and higher heterogeneity.

Heterogeneity among the pooled studies was expressed as an I^2^ value, and the criterion for identifying whether the combined data were more or less heterogeneous was 50%. A random-effects model was performed for significant heterogeneity (I^2^>50%, p≦0.1) due to inconsistencies in the surgical process, anesthesia methods, OLV time, and factors that increase heterogeneity. Sensitivity analysis was also performed to explore possible explanations for the high heterogeneity.

## Results

### Study identification

**Fig 1:**
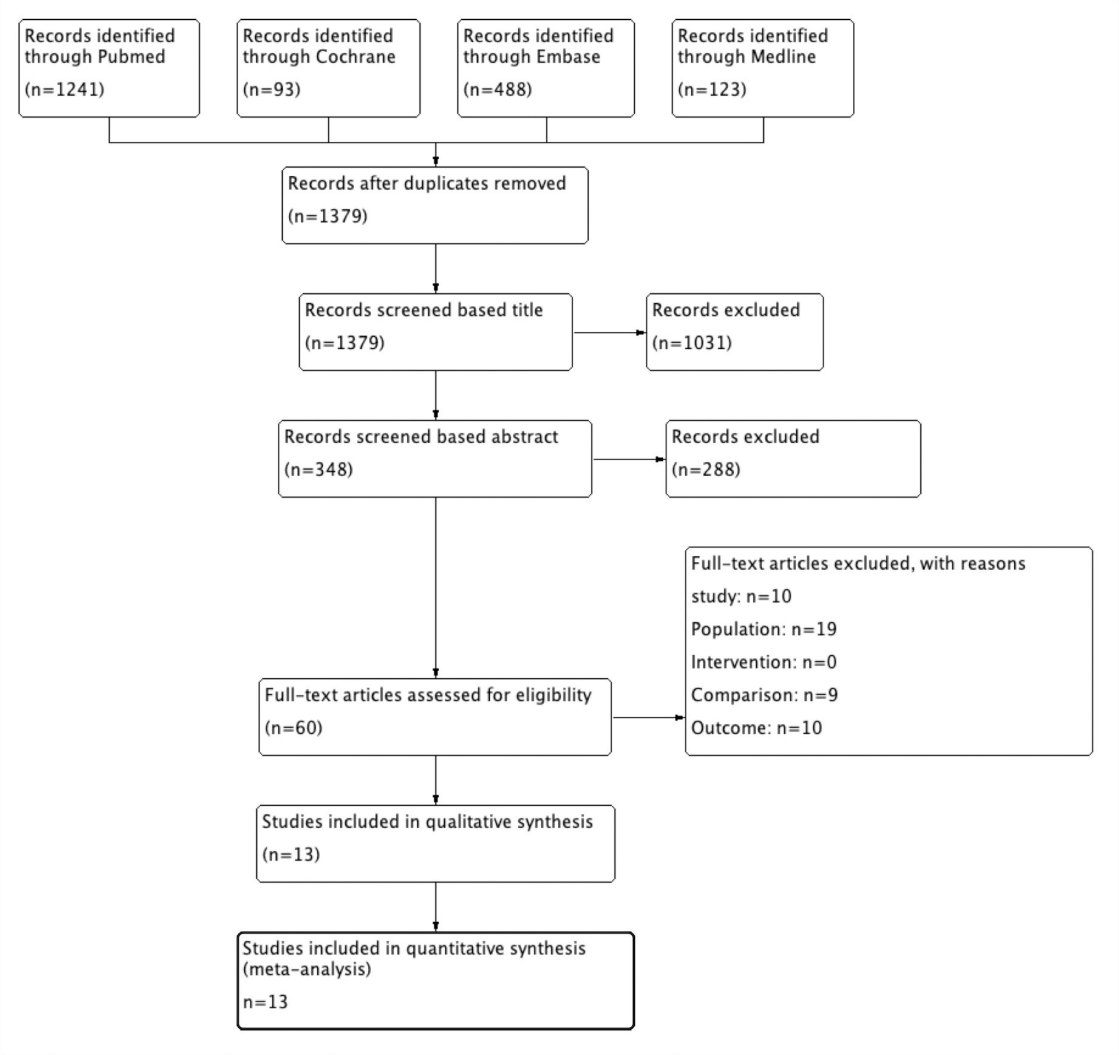
Flow diagram of selecting process.

This search yielded 1945 articles in the initial screening. Based on inclusion and exclusion criteria, 1319 potentially eligible trials were excluded based on title or abstract. Full-text screening excluded 46 studies (10 were not RCTs, 19 did not meet the population criteria, 9 compared intravenous anesthetics with local anesthetics or other agents, and 9 did not report outcomes as previously listed). Finally, 13 studies were included and a meta-analysis was performed [13–25]. The flowchart is shown in Fig 1.

### Study characteristics and quality

**Fig 2:**
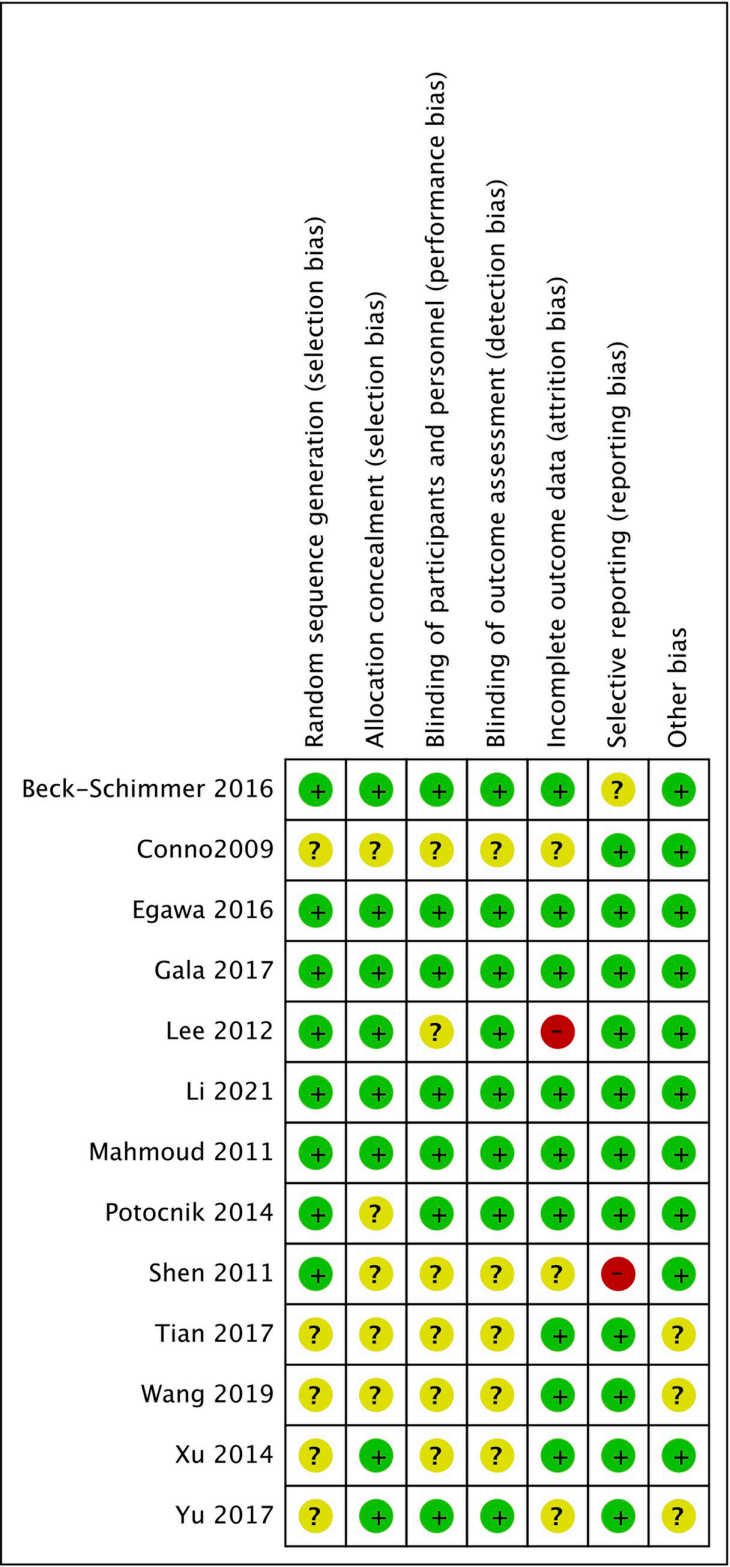
A summary of assessment of risk bias of each RCT.

The main characteristics of the included trials are shown in Table 2. The 13 studies [13–25] included 2522 patients, which published between 2000 and 2021. As showed in Fig 2, 8 of the 13 trials [13, 15–20] showed a low risk of random sequence generation and allocation concealment by describing the randomization method in detail. 6 of the 13 [14, 17, 21–24] did not report details of blinding to participants or outcome assessors, but the impact of lack of blinding on outcomes was considered low. The quality of each outcome was shown in Table 3 by the GRADEpro system. The PPC’s level of evidence was high, and the level of evidence for major complications, 30-day mortality, and length of hospital stay was moderate. However, the level of evidence for MMSE score was low.

**Table 2:** Trial characteristics.

Trial	Surgery	Intervention(n = 1263)		Control(n = 1259)		OLV strategy	Outcome
		Induction	Maintenance	Induction	Maintenance		
Beck-Schimmer 2016 [13]	Lung surgery	Desflurane(n = 230)		Propofol(n = 230)		Vt ^c^ 4–6 ml/kg FIO_2_^d^ 0.6–1.0 PEEP ^e^ 5cmH_2_O	Complications (Clavien-Dindo classification), Hospital stay
		Etomidate (0.3–0.5mg/kg)	Desflurane (end-tidal concentrations of 4.5–7%)	Etomidate (0.3–0.5 mg/kg)	Propofol TCI ^b^ (2-6ug/ml)		
Conno 2009 [14]	Lung surgery	Sevoflurane (n = 27)		Propofol(n = 27)		Vt ^c^ 6-7ml/kg FiO_2_^d^ 1.0	PPCs, Hospital death
		Propofol (1.5–2.5mg/kg)	sevoflurane (1 MAC^a^)	Propofol TCI ^b^(3-5ug/ml)	Propofol TCI ^b^ (1 MAC^a^)		
Gala 2017 [15]	Lung resection surgery	Sevoflurane(n = 86)		Propofol(n = 88)		Vt ^c^ 6ml/kg FiO_2_^d^ 0.6–1.0 PEEP ^e^ 5cmH_2_O	Complications (Clavien-Dindo classification), PPCs, Mortality, Hospital stay
		Propofol (2-3mg/kg)	Sevoflurane (BIS ^f^ 40–60)	Propofol (2-3mg/kg)	Propofol (BIS ^f^ 40–60)		
Egawa 2016 [16]	Lung surgery	Sevoflurane(n = 72)		Propofol(n = 72)		Vt ^c^ 5-6ml/kg FiO_2_^d^ 1.0 PEEP ^e^ 4-5cmH_2_O	MMSE score
		Propofol (1-2mg/kg)	Sevoflurane (BIS ^f^ 40–60)	Propofol TCI ^b^ (3-4ug/ml)	Propofol (BIS ^f^ 40–60)		
Lee 2012 [17]	Ivor Lewis operation	Sevoflurane(n = 24)		Propofol(n = 24)		Vt ^c^ 6ml/kg FIO_2_^d^ (achieve oxygen saturation >95%) PEEP ^e^ 5cmH_2_O	Hospital complications, PPCs, Hospital death, Hospital stay
		Thiopental (4–5 mg/kg)	Sevoflurane (end-tidal concentrations of 1–2.5%)	Propofol (BIS ^f^ 30–50)	Propofol (BIS ^f^ 30–50)		
Li 2021 [18]	Lung surgery	Sevoflurane(n = 169)		Propofol(n = 167)		Vt ^c^ 6ml/kg FiO_2_^d^ 0.4–0.5 PEEP ^e^ 5-8cmH_2_O	Complications (Clavien-Dindo classification), PPCs, Death,
		Propofol (1.5–2.5mg/kg)	Sevoflurane (BIS ^f^ 40–60)	Propofol (1.5–2.5mg/kg)	Propofol (BIS ^f^ 40–60)		
Mahmoud 2011 [19]	Lung surgery	Isoflurane(n = 25)		Propofol(n = 25)		Vt ^c^ 10ml/kg FiO_2_^d^ 0.8–1.0 PEEP ^e^ 5cmH_2_O	PPCs, 30-mortality, Hospital stay
		Propofol (1.5–2 mg/kg)	Isoflurane (1MAC^a^)	Propofol (1.5–2 mg/kg)	Propofol (4-6mg /kg/h)		
Potočnik 2014 [20]	Thoracic surgery	Sevoflurane(n = 17)		Propofol(n = 19)		Vt ^c^ 4ml/kg FiO_2_^d^ 0.6–0.7 PEEP ^e^ 3 cmH_2_O	PPCs, Hospital death
		Sevoflurane (6%)	Sevoflurane (2–2.5%)	Propofol (1.5–2.0 mg/kg)	Propofol (4–6 mg/kg/h)		
Shen 2011 [21]	Thoracic surgery	Sevoflurane (n = 30)		Propofol(n = 30)		Vt ^c^ 8ml/kg FiO_2_^d^ 0.6	MMSE score
		Sevoflurane (4–6%)	Sevoflurane (0.8–1.2MAC^a^)	Propofol (1.5–2 mg/kg)	Propofol (6-8mg /kg/h)		
Tian 2017 [22]	Lobectomy	Sevoflurane(n = 31)		Propofol(n = 31)		Not reported	Adverse reaction, MMSE score
		Sevoflurane (8%)	Sevoflurane (2%)	Propofol (1mg/kg)	Propofol (6mg/kg)		
Wang 2019 [23]	Lung surgery	Sevoflurane(n = 32)		Propofol(n = 26)		Vt ^c^ 8-10ml/kg FiO_2_^d^ 1.0	MMSE score
		Sevoflurane (6%)	Sevoflurane (1MAC ^a^)	Propofol TCI ^b^ (3ug/kg)	Propofol TCI ^b^ (4ug/kg)		
Xu 2014 [24]	Open-chest thoracotomy	Sevoflurane(n = 20)		Propofol(n = 20)		Vt ^c^ 8ml/kg FiO_2_^d^ 1.0	Complications, PPCs, Hospital death, Hospital stay
		Sevoflurane (8%)	Sevoflurane (BIS ^f^ 40–60)	Propofol TCI ^b^ (6ug/ml)	Propofol (BIS ^f^ 40–60)		
Yu 2017 [25]	Thoracic surgery	Sevoflurane(n = 500)		Propofol(n = 500)		Vt ^c^ 8ml/kg	MMSE score
		Sevoflurane (2–4%)	Sevoflurane (BIS ^f^ 45–55)	Propofol (2mg/kg)	Sevoflurane (BIS ^f^ 45–55)		

MAC^a^: minimum alveolar concentration; TCI ^b^: target controll infusion; Vt ^c^: tidal volume; FiO_2_^d^: Fraction of inspiration O_2_; PEEP ^e^: positive end expiratory pressure; BIS ^f^: bispectral index

**Table 3:** The details of GRADE evidence among each outcome.

Certainty assessment							Number of patients		Effect		Certainly	Importance
Studies, n	Study design	Risk of bias	Inconsistency	Indirectness	Imprecision	Other comsiderations	Inhaled anesthetics	Propofol	Relative(95%CI)	Absolute(95%CI)		
Major complications												
5	randomised trials	not serious	not serious	not serious	serious	none	60/542 (11.1%)	74/541 (13.7%)	OR 0.78 (0.54 to 1.18)	27 fewer per 1,000 (from 58 fewer to 21 more)	⨁⨁⨁◯ Moderate	
PPCs												
7	randomised trials	not serious	not serious	not serious	not serious	none	80/381 (21.0%)	113/382 (29.6%)	OR 0.62 (0.44 to 0.87)	89 fewer per 1,000 (from 140 fewer to 28 fewer)	⨁⨁⨁⨁ High	
MMSE scores												
5	randomised trials	serious	serious	not serious	not serious	none	665	659	-	SMD 1.94 SD lower (4.87 lower to 0.99 higher)	⨁⨁◯◯ Low	
Mortality(30-days)												
5	randomised trials	not serious	serious	not serious	not serious	none	3/335 (0.9%)	4/335 (1.2%)	OR 0.79 (0.03 to 18.00)	2 fewer per 1,000 (from 12 fewer to 167 more)	⨁⨁⨁◯ Moderate	
Length of hospital stay												
5	randomised trials	not serious	serious	not serious	not serious	none	385	387	-	SMD 0.05 higer (0.29 lower to 0.39 higher)	⨁⨁⨁◯ Moderate	

PPCs, postoperative pulmonary complications; MMSE, mini-mental state examination; CI, confidence interval; OR, odds ratio; SMD, standard mean difference.

### Primary outcome: major postoperative complications

**Fig 3:**
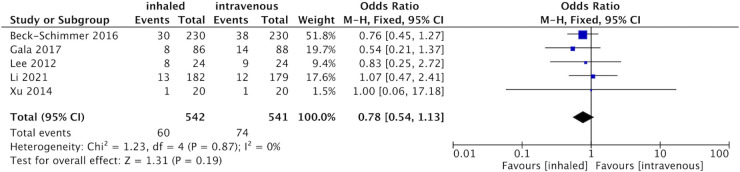
Forest plot for the number of the major postoperative complications between inhaled and propofol groups.

As mentioned earlier, major complications mean that patients require more intensive care: Five studies [13, 15, 17, 18, 24] evaluated major complications in 1083 patients who underwent OLV. Moreover, the overall incidence of major complications after OLV was 12.37%. However, in our evidence-based analysis, compared to the propofol group, inhaled anesthetics were not associated with a lower incidence of major complications after OLV (OR 0.78, 95% CI 0.54 to 1.13, p = 0.19; I^2^ = 0, Fig 3).

### Secondary outcomes

#### PPC

**Fig 4:**
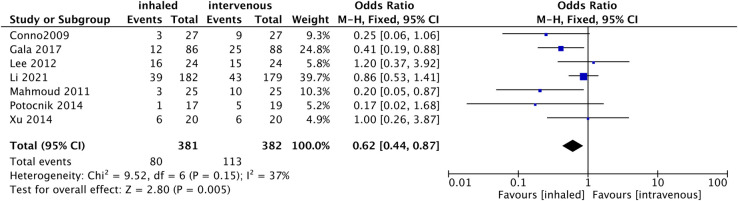
Forest plot for the number of postoperative pulmonary complications between inhaled and propofol groups.

Seven RCTs [14, 15, 17–20, 24] compared the effect of propofol and inhaled anesthetics on PPC in 763 patients with OLV. Pooled data showed that the incidence of postoperative PPC was 20.9% in the propofol group and 29.6% in the inhaled anesthetic group. And a fixed effects model showed that inhaled anesthetics were less heterogeneous and significantly reduced the number of patients who developed PPC compared to propofol (OR 0.62, 95%CI 0.44 to 0.87, p = 0.005; I^2^ = 37%, Fig 4).

#### Postoperative MMSE scores

**Fig 5:**
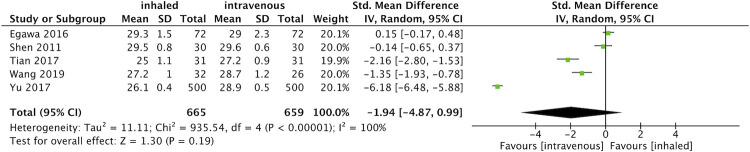
Forest plot for the postoperative MMSE scores between inhaled and propofol groups.

As shown in Fig 5, five RCTs [16, 21–23, 25] estimated postoperative cognitive function after OLV with MMSE scores in 1324 patients. They found little effect of anesthetic type on MMSE scores (SMD -1.94, 95% CI -4.87 to 0.99, p = 0.19; I^2^ = 100%). Due to the very high heterogeneity, further sensitivity analysis and HKSJ methods were performed to reinforce the results. However, removing individual trials did not yield the original heterogeneity, and the HKSJ method reached the same conclusion as before (SMD -1.94, 95%CI -5.11 to 1.23, p = 0.16).

#### Length of hospital stay

**Fig 6:**
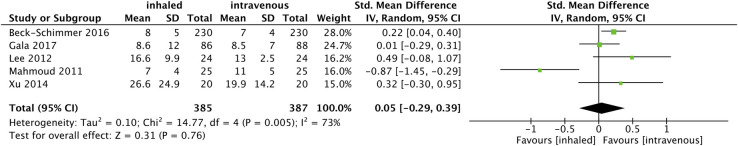
Forest plot for the length of hospital stay between inhaled and propofol groups.

For the length of hospital stay, data were extracted from 5 trials [13, 15, 17, 19, 24] and 772 patients. Fig 6 shows that there was no significant difference at all in the length of hospital stay between different anesthetic types (SMD 0.05, 95% CI -0.29 to 0.39, p = 0.76; I^2^ = 73%, Fig 6). Sensitivity analysis detected that Mahmoud, et al. [19] contributed to the overall heterogeneity. Pooled data excluding this study confirmed that the propofol group had a significantly shorter hospital stay than the inhalation group (SMD 0.19; 95% CI 0.05 to 0.34, p = 0.01; I^2^ = 0). However, the HKSJ method, excluding Mahmoud, et al. led to the conclusion that anesthetics were not related to the length of hospitalization, depicting the instability of the above results (SMD 0.19, 95% CI -0.04 to 0.42, p = 0.07).

#### 30-days mortality rate

**Fig 7:**
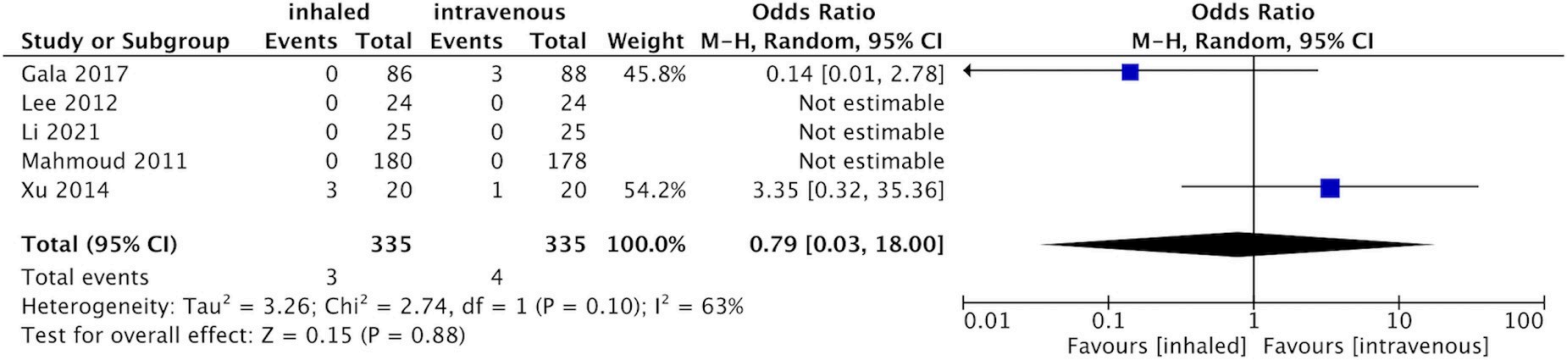
Forest plot for the number of 30-day death between inhaled and propofol groups.

Two of five studies [15, 17–19, 24] were designed to evaluate mortality within 30 days postoperatively. The results showed that 3 patients in the inhalation group and 4 patients in the propofol group died within 30 days postoperatively. Due to the small number of papers, we found no difference in mortality within 30 days between the two groups (SMD 0.79, 95% CI 0.03 to 18, p = 0.88; I^2^ = 63%, Fig 7).

## Discussion

This analysis included 13 eligible trials [13–25] of 2522 patients undergoing OLV and described substantial evidence, evaluated with the GRADEpro system, that compared to propofol, inhaled anesthetics carry less risk of PPC. However, there were no significant differences in major complications, postoperative MMSE scores, length of hospital stay, or 30-day mortality by anesthetic type.

In a meta-analysis by Uhlig, et al. [6], in cardiac surgery, general anesthesia with inhaled anesthetics was associated with decreased major complications and mortality, likely due to the cardioprotective effects of volatile anesthetics through coronary vasodilation and decreased stress response [26]. Similarly, Uhlig, et al. [6] concluded that in noncardiac surgery, inhaled anesthetics appear to offer little advantage over intravenous anesthetics in major complications, mortality, and length of hospital stay. However, previous studies have shown that the anti-inflammatory effects of volatile anesthetics can affect other organs such as the lungs, brain, kidneys, and liver [27–29]. For patients undergoing noncardiac surgery (e.g., thoracic, vascular, and abdominal surgery), major complications, length of hospital stay, and mortality may be related more to the type of surgery, patient characteristics, and standardized surgical procedures than to the type of anesthetic. Therefore, the systematic organ protection of inhaled anesthetics was rare for the patients undergoing OLV studied in this study.

To our knowledge, inhaled anesthetics inhibit hypoxic pulmonary vasoconstriction (HPV) and cause hypoxemia when used at a minimum alveolar concentration greater than one during OLV [30]. However, Prakash [31] observed that volatile agents act directly on bronchial smooth muscle, contributing to bronchodilation, and that Cdyn acts to a greater extent at lower pressures during OLV compared to propofol. Thus, there are both advantages and disadvantages of inhaled anesthetics on lung function during OLV.

With regard to in vitro [32] or in vivo [33] inflammatory responses, inhaled anesthetics were found to significantly suppress the inflammatory response to lung injury, contributing to immunomodulatory and organ protective effects. In clinical surgery involving OLV, inhaled anesthetics were also found to exert anti-inflammatory effects by acting on cytokine responses, ischemia-reperfusion, and oxidative stress [15, 34]. A meta-analysis also concluded that compared to intravenous anesthesia, inhaled anesthesia could reduce the alveolar inflammatory response, but has no significant effect on the systemic inflammatory response in the interim [10]. This may contribute to the finding that inhalational agents are more effective in reducing the occurrence of PPC rather than other systemic complications.

From the reports of the International Working Group on Perioperative Neurotoxicity, little evidence has been detected regarding which anesthetic is preferred for postoperative cognitive function in general anesthesia [35]. Studies have demonstrated that cerebral oxygen saturation is associated with postoperative cognitive dysfunction [36], and OLV is also associated with a definite decrease in partial pressure of oxygen compared to baseline [37]. Furthermore, trials have shown that during the first 30 minutes after OLV, the oxygenation index is higher in the intravenous anesthetic group compared to the inhaled anesthetic group [8]. However, consistent with the recommendations of the working group, we found that postoperative cognitive function screening (MMSE score) after OLV was comparable between the two groups. This may be because MMSE screening is inadequate to measure cognitive function, and postoperative cognitive dysfunction may last for weeks or months, and the follow-up period of the included clinical trials was not long enough. More importantly, desaturation, which could offset differences in the effects of anesthetics on cognitive function, was rare in all participants.

The analysis revealed several limitations. First, all trials did not systematically apply the Clavien-Dindo score in the evaluation of major complications and analyzed complications on a scale of 0 to 5 severity. Due to the limited articles, postoperative events were defined as those requiring more intensive treatment in order to reduce the risk of bias as much as possible. Second, only two trials reported mortality. Mortality rates are relatively low and are more influenced by multiple factors than by anesthetics alone. Therefore, this conclusion can be used as a reference. Next, some of the data obtained were transferred from the median/range and graphs. Although this is a commonly used method, it may increase the risk of error rates since the data are not entirely original. The language is also limited to English, which may increase the risk of publication bias. Therefore, if researchers doubt the prognostic value of different anesthetics during OLV, as recommended by two Cochrane meta-analyses [3, 7], then higher quality, more extensive trials should be designed and conducted in the future to evaluate expected outcomes.

## Conclusion

In patients with OLV, inhaled anesthetics had a significant protective effect against PPC compared to propofol, but had no effect on major postoperative complications, cognitive function, length of hospital stay, or mortality at 30 days. Further studies are needed to validate this conclusion.

## Abbreviations

OLV: one lung ventilation
PPCs: postoperative pulmonary complications
MMSE: Mini-mental State Examination
